# Supplementary material for: MetaRibo-Seq measures translation in microbiomes
Source: Nat Commun. 2020 Jun 29;11:3268. doi: 10.1038/s41467-020-17081-z (PMC7324362; doi:10.1038/s41467-020-17081-z)
Supplement: Supplementary file 10 — Supplementary Data 7 [file 41467_2020_17081_MOESM10_ESM.zip › File2/Confidence_High_Taxonomy/372010_out.krona.html]

Javascript must be enabled to view this page.

members
magnitude
magnitudeUnassigned
count
unassigned
taxon
rank

372010\_out

1

2
1
superkingdom

1
phylum
1239

91061
1
class

order
1
186826

1300
family
1

1
genus
1301

671232
species group
1

1328
1
species

SRS024015\_contig\_number\_contig-100\_76.93523
